# The optimum economic nitrogen rate of blended controlled-release nitrogen fertilizer for rice in the Chanoyu watershed in the Yangtze River Delta, China

**DOI:** 10.3389/fpls.2023.1144461

**Published:** 2023-04-11

**Authors:** Manman Yuan, Yixiang Sun, Gang Wu, Jiabao Wang, Chuang Liu, Tongtong Tang, Xiangming Zhang, Wenjun Wang, Yudan Jing

**Affiliations:** ^1^ Key Laboratory of Nutrient Cycling, Resources and Environment of Anhui, Institute of Soil and Fertilizer, Anhui Academy of Agricultural Sciences, Hefei, China; ^2^ School of Biological Science and Food Engineering, Chuzhou University, Chuzhou, China

**Keywords:** optimum economic nitrogen rate, nitrogen release, rice, yield, NH3 volatilization, environmental loss

## Abstract

**Introduction:**

The application of controlled-release nitrogen fertilizer (CRN) has become an important production method to achieve high crop yield and ecological safety. However, the rate of urea-blended CRN for rice is usually determined by conventional urea, and the actual rate is still unclear.

**Methods:**

A five-year field experiment was carried out in the Chaohu watershed in the Yangtze River Delta to study rice yield, N fertilizer utilization efficiency (NUE), ammonia (NH3) volatilization and economic benefit under the four urea-blended CRN treatments with a 4:3:3 ratio applied at one time (60, 120, 180, 240 kg/hm2, CRN60, CRN120, CRN180, CRN240), four conventional N fertilizer treatments (N60, N120, N180, N240) and a control without N fertilizer (N0).

**Results and Discussion:**

The results showed that the N released from the blended CRNs could well satisfy the N demand of rice growth. Similar to the conventional N fertilizer treatments, a quadratic equation was used to model the relationship between rice yield and N rate under the blended CRN treatments. The blended CRN treatments increased rice yield by 0.9-8.2% and NUE by 6.9-14.8%, respectively, compared with the conventional N fertilizer treatments at the same N application rate. The increase in NUE in response to applied blended CRN was related to the reduction in NH3 volatilization. Based on the quadratic equation, the five-year average NUE under the blended CRN treatment was 42.0% when rice yield reached the maximum, which was 28.9% higher than that under the conventional N fertilizer treatment. Among all treatments, CRN180 had the highest yield and net benefit in 2019. Considering the yield output, environmental loss, labor and fertilizer costs, the optimum economic N rate under the blended CRN treatment in the Chaohu watershed was 180-214 kg/hm2, compared with 212-278 kg/hm2 under the conventional N fertilizer treatment. The findings suggest that blended CRN improved rice yield, NUE and economic income while decreasing NH3 volatilization and negative environmental outcomes.

## 1 Introduction

Rice provides food for approximately half of the world’s population and is a major source of calories and protein for poor people in Asia and Africa (Muthayya et al., 2015). According to the estimate of the Food and Agriculture Organization of the United Nations, the world population will reach 9 billion by 2050, and food production will need to increase by at least 70% to meet the demand due to population growth (Niggli, 2015). Nitrogen (N) is an essential nutrient for rice growth. High rates of N fertilizer are applied to achieve a high yield of rice. China consumes more than one-third of the world’s total N fertilizer and is currently the largest N fertilizer user in the world (IFA, 2021). The Chaohu watershed in the Yangtze River Delta in China is one of the major rice production areas in China. The maximum application rate of chemical N fertilizer during the rice growing period can reach 300 kg/hm^2^. The N fertilizer utilization efficiency (NUE) of rice was reported to be 39% in this area (Yu and Shi, 2015), which is 20–30 percentage points lower than the levels in developed countries such as Europe and the United States (Lassaletta et al., 2014). Excessive application of N fertilizer not only increases the cost of rice production, reduces NUE, and leads to the eutrophication of water bodies and soil compaction (Congreves et al., 2021), but also causes the volatilization of nitrogen oxides and ammonia (NH_3_) into the air, and these gases have become the main components of haze and reduced atmospheric quality (Wang et al., 2016a). NH_3_ volatilization from agricultural production was estimated to account for more than 50% of global NH_3_ emissions (Gronwald et al., 2018). Therefore, to control the contribution of N loss in paddy fields to environmental emissions, research on nutrient reduction technology in agricultural fields has become a current research hotspot.

To reduce N use and loss, fertilization measures such as deep application, split application, and N fertilizer setback are often used in rice production (Emran et al., 2019; Wang et al., 2019; Ju et al., 2021), but they still present existing problems in actual production. On the one hand, topdressing in a split application is often spread on the soil surface, which can cause nutrient runoff, leaching, and NH_3_ volatilization (Wang et al., 2019; Zhan et al., 2020; Song et al., 2021). On the other hand, due to the rapid development of rice farming intensification and the decrease in the agricultural labor force, rice farmers with large land areas generally face the problems of labor shortages and increasing labor costs (Luo, 2021; Xu et al., 2021). The study of light and simplified fertilizer application technology for rice is important to improve labor efficiency and reduce the cost of fertilizer application.

Controlled-release N fertilizer (CRN) has the advantages of a high synchronization rate between nutrient release and crop nutrient uptake and high fertilizer efficiency, which allows for one-time fertilization of rice, solving the difficult problem of topdressing rice, decreasing N loss, improving NUE, and reducing environmental risks (Chen et al., 2020; Guo et al., 2019; Li et al., 2015; Zhang et al., 2018). It was found that different types of CRNs could reduce N loss in paddy fields compared with the split application of conventional N fertilizer at the same N application rate, and the effect was better when blended CRN and conventional N fertilizer were applied at different ratios (Lyu et al., 2021a). However, the current application rates of blended CRN are mainly determined by the application rate of urea (Lyu et al., 2021a), while the appropriate rate of blended CRN to further reduce the environmental risk based on ensuring rice yield is still unclear.

Therefore, based on five consecutive years of rice field location trials in the Chaohu watershed, this study compared the differences in rice yield, NUE, and NH_3_ volatilization between the split application of urea and the one-time application of blended CRNs and urea under different N rates. The optimum economic N application rate for rice was evaluated using an environmental and economic evaluation method that considered the reduction in the N rate while guaranteeing a high rice yield. This study is expected to provide a scientific basis for the determination of an ecologically suitable fertilization technology for rice in the Chaohu watershed.

## 2 Materials and methods

### 2.1 Experimental materials

**Figure 1:**
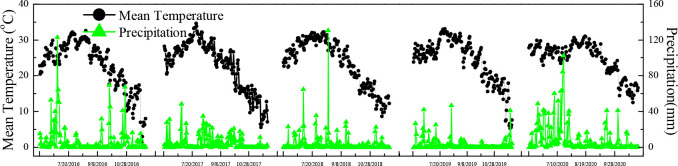
Mean temperature and precipitation in the rice season from 2016 to 2020.

Field experiments were conducted over five years from 2016 to 2020 in Zhonghan village, Hefei city, Anhui Province, located in the Chaohu watershed in the Yangtze River Delta, China (N 31°39′14″, E117°46′34″), with a long history of rice cultivation and a northern subtropical monsoon climate (**Figure 1**; **Figure S1**). The annual average temperature and precipitation are 16.1°C and 1,030 mm, respectively. The soil type is gleyed paddy soil, and the main initial properties of the top-layer soil (0–20 cm) were as follows: pH, 7.1 (1:2.5, soil/water); total N, 1.9 g/kg; available phosphorus, 9.0 mg/kg; available potassium, 198.8 mg/kg; and organic matter, 29.1 g/kg.

**Figure 2:**
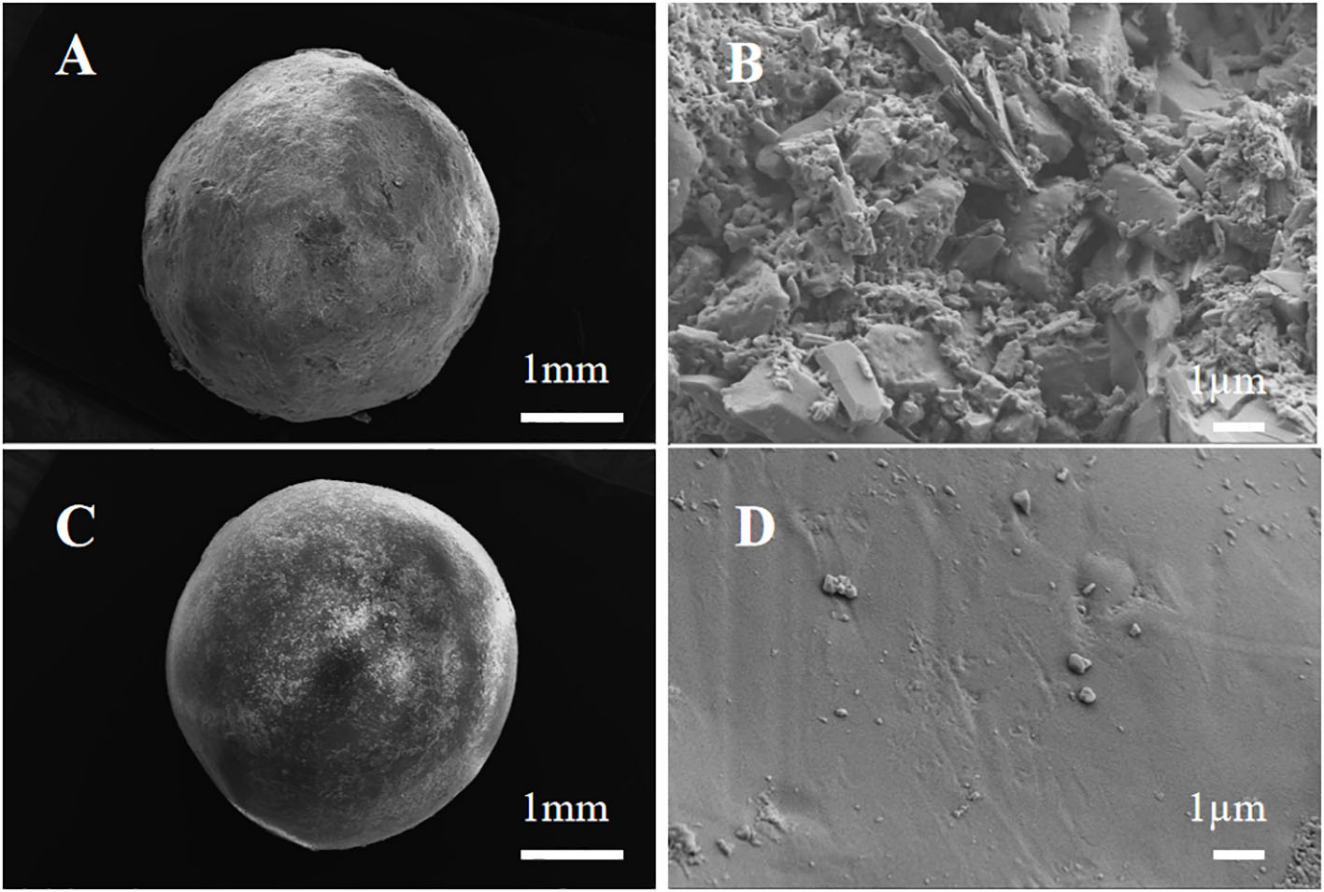
Surface scanning electron microscopy (SEM) of controlled-release N fertilizers (CRNs) with release periods of 40 d **(A, B)** and 90 d **(C, D)**.

**Figure 3:**
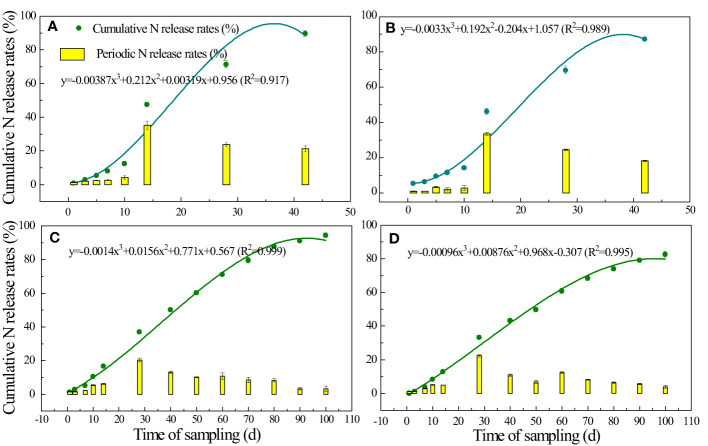
Cumulative N release rates (curves) and periodic N release rates (bars) of CRNs with periods of 40 d **(A, B)** and 90 d **(C, D)** incubated in paddy fields **(A, C)** and water (25°C) **(B, D)** in 2019.

The controlled-release nitrogen fertilizer (CRN, 44.5% N) was obtained from Anhui Moith Agricultural Technology Co., Ltd. (Anhui, China), the center of which was a urea pellet coated with polyurethane. In this experiment, the periods of CRN were 40 and 90 days, represented by CRN1 and CRN2, respectively. The N release durations of CRN1 and CRN2 in 25°C water and paddy fields were measured at 42 and 100 days in 2019 (**Figures 2A–D**, **3A–D**).

Conventional N, phosphorus, and potassium fertilizers were applied in the form of urea (46% N), heavy granular superphosphate (42% P_2_O_5_), and potassium chloride (60% K_2_O).

### 2.2 Experimental design

The experiment was conducted in a split-plot block design: the main plot was the type of N fertilizer and its application method, and the subplot was the amount of nitrogen fertilizer. There were nine treatments and three replications. The plot area was 30 m^2^ (5 m × 6 m). The specific treatments were as follows: control without N fertilizer (N0); four blended CRN treatments with a 4:3:3 ratio [60 kg/hm^2^ (CRN60), 120 kg/hm^2^ (CRN120), 180 kg/hm^2^ (CRN180), and 240 kg/hm^2^ (CRN240)] applied at one time but with conventional N fertilizer, CRN1 and CRN2; and four conventional N fertilizer treatments [60 kg/hm^2^ (N60), 120 kg/hm^2^ (N120), 180 kg/hm^2^ (N180), and 240 kg/hm^2^ (N240)] in accordance with a 5:3:2 ratio of the N rate with three applications of basal, tillering, and panicle fertilizer. Phosphorus (P_2_O_5_) and potassium (K_2_O) fertilizers were applied at one time as basal fertilizers, at rates of 90 and 75 kg/hm^2^, respectively.

The rice variety and timing of planting and management operations in this study are shown in **Table 1**. The spacing of transplanted rice was 0.30 m (length) by 0.13 m (width).

**Table 1:** Rice variety and timing of the planting and management operations.

Year	Rice variety	Sowing	Transplanting	Basal fertilization	Tillering topdressing	Heading topdressing	Harvest
2016	Huang hua zhan	5/26	6/29	6/29	7/7	8/10	10/22
2017	Chaoyou 1000	5/12	6/14	6/14	6/24	7/23	10/1
2018	Chaoyou 1000	5/20	6/21	6/21	7/3	8/1	10/9
2019	Chaoyou 1000	5/25	6/29	6/29	7/6	8/2	10/8
2020	Chaoyou 1000	5/20	6/22	6/22	6/30	7/29	10/10

### 2.3 Sample processing and measurements

NH_3_ volatilization was measured by the semi-open static cylinder system (Kissel et al., 1977; Jantalia et al., 2012; Feng et al., 2017), which was a plexiglass enclosure 20 cm in diameter and 15 cm in height with ventilation holes at the top. Volatilized NH_3_ was absorbed with a 20 g/L boric acid solution and titrated with standard acid (C_1/2H2SO4_ = 0.020678 mol/L) at the end of each collection. Air extraction was performed from 9:00 to 11:00 am and from 14:00 to 17:00 pm each day after fertilization. The air exchange frequency was 16 to 20 times/min. The amount of NH_3_ volatilized throughout the day was calculated based on this average value (Yu et al., 2013). NH_3_ volatilization was determined continuously after fertilizer was applied until it was not detected.

At maturity, the tiller number of rice per square meter was investigated in each subplot. Based on the mean tiller number per hill, rice from three representative hills was selected and destructively sampled. The samples were separated into plants (including leaves and stems) and panicles. Then, the samples were oven-dried at 105°C for 2 h and then at 70°C for 72 h, and the dry matter was measured. The N concentrations of the samples were determined by the Kjeldahl method (Bremner & Mulvaney, 1982). Grain yield was manually determined based on all plants within an area of 5 m × 3 m within each subplot after the grain moisture content at the time of harvest was adjusted to 0.14 g/g fresh weight.

The release curves of the CRNs with release periods of 40 and 90 days were determined both in paddy fields and in the laboratory using the method reported by Geng et al. (2015), as described below.

CRN1 and CRN2 (10 g) were placed in a nylon bag with an aperture of 0.15 mm and sealed. When rice was transplanted in 2019, the fertilizer materials were buried in the paddy soil without fertilizer near the experimental site, at a depth of 10 cm. In the laboratory, the samples were placed in a 300-ml plastic bottle to which 250 ml of laboratory-pure water was added, and then the bottle was sealed and placed in a biochemical thermostatic incubator at 25 °C. Samples of CRN1 were collected every 24 h and then on the 3rd, 7th, 10th, 14th, 28th, and 42nd days, respectively. Samples of CRN2 were collected at 24 h on the 3rd, 7th, 10th, 14th, 28th, 40th, 50th, 60th, 70th, 80th, 90th, and 100th days, respectively. Three bags of each fertilizer were collected each time. For the samples of paddy soil, after the cultivation bag was opened, all the soil and fertilizer were transferred to a sieve with an aperture of 1 mm and slowly washed with laboratory-pure water to clean up the soil. Then, the fertilizer film was removed. The fertilizer that was removed was filtered into a 250-ml volumetric flask. The N concentrations of the fertilizer solution were determined by the Kjeldahl method (Bremner & Mulvaney, 1982). For the samples in the laboratory, during sampling, the bottle was inverted up and down three times to ensure a homogeneously mixed solution. Subsequently, the fertilizer solution was transferred into another bottle to determine the N concentration, after which 250 ml of laboratory-pure water was added to the bottle containing the fertilizer bag, and then the bottle was sealed and placed in a biochemical thermostatic incubator at 25 °C for further incubation.

The surface morphology of the CRNs was determined by a scanning electron microscope (SEM, ZEISS Sigma 300 EDS: Model Smart EDX, Britain).

### 2.4 Calculation and statistics

NH_3_ volatilization flux was calculated according to the following equation (Jantalia et al., 2012; Feng et al., 2017).


(1) $$F=\frac{V\times C\times 0.014\times 12\times 10^{-3}}{\text{π}\times r^{2}\times 10^{-4}}$$

where *F* is the NH_3_ volatilization flux (kg/hm^2^/d); *V* is the volume of standard acid for titration (mL); 10^−3^ is the conversion of mL to L; *C* is the calibration concentration of standard acid for titration (mol/L); 0.014 is the relative atomic mass of N atoms (kg/mol); 10^4^ is the conversion of m^2^ to hm^2^; *r* is the radius of the airtight chamber (m); and 12 is the conversion to NH_3_ volatilization flux over a one-day period.

Cumulative NH_3_ emissions were calculated according to the following equation (Jantalia et al., 2012; Feng et al., 2017).


(2) $$E_{{\text{NH}}_{3}}=\sum_{i=1}^{n}\left(\frac{F_{i}+F_{i+1}}{2}\right)\times \left(t_{i+1}-t_{i}\right)$$

*E*_NH_3__ represents the cumulative NH_3_ emissions (kg/hm^2^); *Fi* is the NH_3_ volatilization flux (kg/hm^2^/d) on sampling day *i*; *i* represents the *i*th sampling day; and *t_i + 1_* − *t*represents the time interval (d) between two sampling measurements.

The NH_3_ emission factor (*EF*_NH_3__ .) as calculated according to the following equation (Huang et al., 2016).


(3) $$EF_{{\text{NH}}_{3}}=\frac{\;\;E_{{\text{NH}}_{3}-\text{fertilizer}}\;-\;E_{{\text{NH}}_{3}-\text{N}0}}{F_{\text{N}}}$$

  *E*_NH_3_−fertilizer_ represents the cumulative NH_3_ emissions under N application (kg/hm^2^),  *E*_NH_3_−N0_ presents the cumulative NH_3_ emissions under the N0 treatment (kg/hm^2^), and *F*_N_ is the N application rate (kg/hm^2^).

Yield-scale NH_3_ emissions [kg/t(grain)] were calculated according to the following equation (Huang et al., 2016).


(4) $$E_{NH}{\;}_{3}yield-scale=\frac{E_{{\text{NH}}_{3}}}{Y}$$

*E*_NH_3__ represents the cumulative NH_3_ emissions (kg/hm^2^), and *Y* is the rice grain yield under each treatment (t/hm^2^).

Nitrogen use efficiency (NUE) was calculated according to the following equation (Sun et al., 2012):


(5) $$NUE\left(\%\right)=\frac{U_{\text{N}}-U_{0}}{F_{\text{N}}}$$

*U_N_* is rice grain and plant N accumulation under N application (kg/hm^2^); *U*_0_ is grain and plant N accumulation under N0 treatment (kg/hm^2^); *F*_N_ is the N application rate (kg/hm^2^).

According to the Eco-indicator 95 transformation procedure, 1 kg NH_3_ volatilization flux is equivalent to the eutrophication effect of 0.33 kg PO_4_^3−^ and the acid rain effect of 1.88 kg SO_2_ (Goedkoop, 1995). However, 4% of the Chaohu watershed is natural wetlands (rivers, lakes, marshes, etc.) (Wu et al., 2020). Therefore, 96% of NH_3_ volatilization will cause acidification through wet and dry deposition on the soil surface. The NH_3_ deposited on the water surface has a eutrophication effect on the water body. The marginal environmental loss (*M*_1_, yuan/hm^2^) for the acid rain effect and the marginal environmental loss (*M*_2_, yuan/hm^2^) for the eutrophication effect of NH_3_ volatilization from N application can be expressed by the following equations (Xia and Yan, 2012):


(6) $$M_{1}=\;96\%\;\times \;1.88\;\times E_{{\text{NH}}_{3}}P_{a}\times \;17/14$$

(7) $$M_{2}=\;4\%\times \;0.33\;\times E_{{\text{NH}}_{3}}P_{e}\times \;17/14$$

In the equation, 1.88 is the conversion factor of the acid rain effect of 1 kg cumulative NH_3_ emission equivalent SO_2_; *P*_a_ is the acid rain loss caused per kg SO_2_ (yuan/kg), which was 5 yuan/kg in this study; *P*_e_ is the loss of the eutrophication effect caused per kg PO_4_^3−^ (yuan/kg), which was 3.88 yuan/kg in this study; *E*_NH_3__ represents the cumulative NH_3_ emissions (kg/hm^2^); and 17/14 is the conversion factor of N to NH_3_.

The environmental loss caused by NH_3_ volatilization (*M*_loss_, yuan/kg) was calculated according to the following equation.


(8) $$M_{loss}=M_{1}+M_{2}$$

The net benefit from rice planting in the Chaohu watershed (*M*_net_, yuan/kg) was calculated according to the following equation.


(9) $$M_{net}=M_{Y}-M_{L}-M_{F}-M_{loss}$$

*M*_Y_ is the output value of rice yield (yuan/hm^2^), and the price of rice sold in 2019 was 2.5 yuan/kg; *M*_L_ is the labor cost of rice fertilization, and the labor cost of rice fertilization per event was 900 yuan/hm^2^; and *M*_F_ is the fertilizer cost. The prices of urea, CRN, potassium, and phosphorus fertilizer were 2.0, 2.8, 2.0 and 0.6 yuan/kg, respectively, in 2019; *M*_loss_ is the environmental loss of NH_3_ volatilization.

### 2.5 Statistical analysis

SPSS 22.0 software (IBM Corp., Armonk, NY, USA) was used to perform multivariate analyses based on a general linear model. Pearson’s correlation was used to analyze the relationships between parameters. Origin 2022 (OriginLab Corp., Northampton, MA, USA) was used to plot the figures.

## 3 Results

### 3.1 Release characteristics of CRNs

Scanning electron microscopy images clearly showed the coating materials and fertilizer particles of CRN1 and CRN2 (**Figures 2A–D**). When magnified 44 times, the surfaces of both CRN1 and CRN2 were smooth (**Figures 2A, C**). When magnified 20,000 times, the surface of CRN2 was still smooth. However, the surface of CRN1 with a N release period of 40 days was somewhat loose compared with that of CRN2 (**Figures 2B, D**).

The N release curves of CRN1 and CRN2 showed a steady release rate in the paddy field and 25°C water, which fit cubic binomial curves (**Figures 3A–D**). The cumulative N release rate of CRN1 in the paddy field was 89.7% of the supplemental N released within 40 days (**Figure 3A**), while that of CRN2 was 91.2% within 90 days (**Figure 3C**). The N release characteristics in 25°C water were slightly slower (**Figures 3B, D**), with 86.7% of N released in 40 days for CNR1 and 87.4% of N released in 90 days for CNR2; the maximum periodic N release rate was approximately 1/3 of their release periods. In the paddy field, the cumulative N release rate of CRN1 was 33.5% in 14 days, and that of CRN2 was 22.3% in 28 days. The results were similar in water.

### 3.2 Rice yield

**Figure 4:**
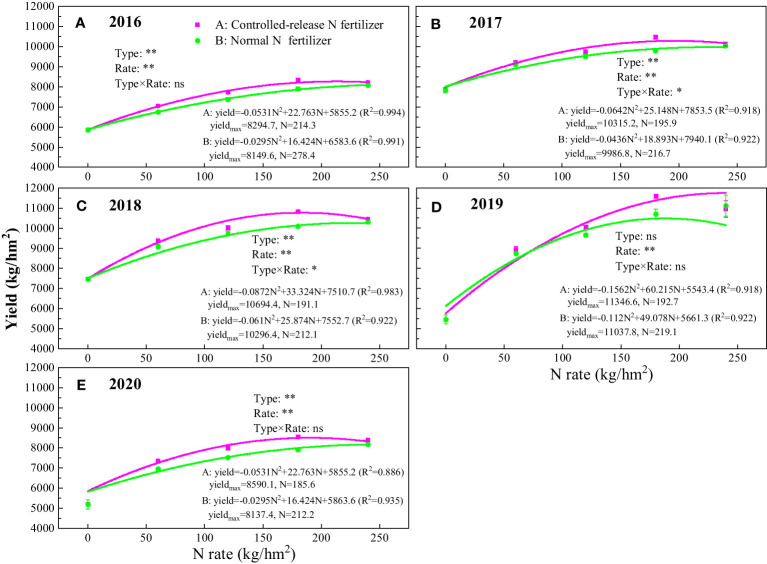
Rice yield under the blended CRN treatments (red square) and conventional N fertilizer treatments (green circle) with applied N rates of 0, 60, 120, 180, and 240 kg/hm^2^ from 2016 to 2020 **(A–E)**. Statistically significant differences (**P<0.01; *P<0.05) and no significant differences (P >0.05, ns) are shown. Values are the means ± SEs (n = 3).

The rates and types of N fertilizers and their application methods had a significant effect on rice yield. The yield results for five consecutive years from 2016 to 2020 showed consistent trends. The rice yields under the N application treatments were significantly higher than those under N0 (*P<*0.01) (**Figures 4A–E**). Under the blended CRN treatments, yields increased with increasing N application rates of 60–180 kg/hm^2^. However, the yield under CRN240 decreased by 1.5%–5.8% compared with that under CRN180 from 2016 to 2020. Under the conventional N fertilizer treatments, yields increased with increasing N application rates of 60–240 kg/hm^2^. At the same N application rate of 60 to 180 kg/hm^2^, the yield was significantly higher under the blended CRN treatments than under the conventional N fertilizer treatments (*P<*0.05). The results of the five-year experiment showed that yield increased by 0.9%–4.3% under CRN60 compared to N60, by 2.4%–6.3% under CRN120 compared to N120, and by 5.6%–8.2% under CRN180 compared to N180. In contrast, the yields of CRN240 and N240 were not significantly different (*P >*0.05), with an average increase of 0.9% over 5 years.

**Figure 5:**
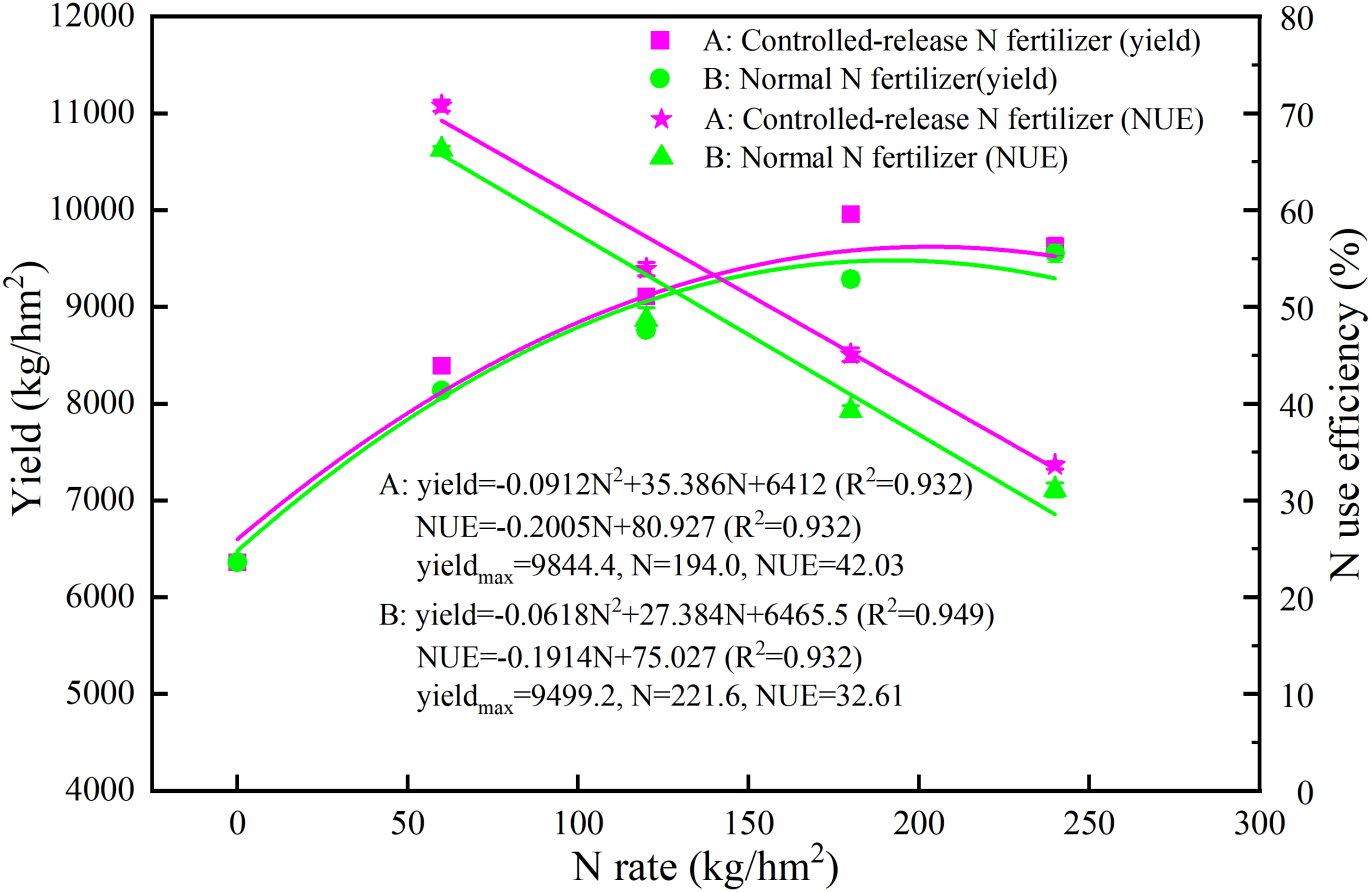
Average yield and N use efficiency (NUE) of rice from 2016 to 2020 under the blended CRN treatments (red graph) and conventional N fertilizer treatments (green graph) with applied N rates of 0, 60, 120, 180, and 240 kg/hm^2^.

The relationship between rice yield and the N application rate was modeled by a quadratic equation (**Figures 4A–E**). As simulated by the equation, the highest yield of rice in the five years under the blended CRN treatments was 8,295–11,347 kg/hm^2^, and the corresponding N application rate was 191–214 kg/hm^2^ (**Figures 4A–E**). The average highest yield was 9,844 kg/hm^2^ with an N application rate of 194 kg/hm^2^ under CRN treatments (**Figure 5**). Under conventional N fertilizer treatments, the highest yield of rice in the five years was 8,137–11,038 kg/hm^2^, corresponding to 212–278 kg/hm^2^ of N applied (**Figures 4A–E**). The average highest yield was 9,499 kg/hm^2^ with an N application rate of 222 kg/hm^2^ (**Figure 5**). The highest yield under the blended CRN treatments increased by 1.8%–5.6% in the five years compared with that under the conventional N fertilizer treatments, with an average increase of 3.6%, and the corresponding N application rate decreased by 21–60 kg/hm^2^, with an average decrease of 12.5%.

### 3.3 Rice NUE

The trend of the rice NUE was consistent for five consecutive years from 2016 to 2020, with NUE showing a negative correlation with the N application rate (**Figure 5**; **Table 2**). The NUE of the blended CNR treatments was significantly higher than that of the conventional N fertilizer treatments at the same N application rate (*P<*0.01). The increases were 0.5%–7.8%, 8.9%–15.4%, 10.3%–16.7%, and 4.9%–11.5% at N rates of 60, 120, 180, and 240 kg/hm^2^ over 5 years, with average increases of 6.9%, 10.7%, 14.8%, and 8.2%, respectively (**Table 2**). According to the simulation based on the quadratic equation, when the average rice yield was the highest over the five years, the NUE of the blended CNR treatments was 42.0% and that of the conventional N fertilizer treatments was 32.6%, which was an increase of 28.9% compared with the NUE under the conventional N fertilizer treatments (**Figure 5**).

**Table 2:** N fertilizer utilization efficiency (NUE) of rice under different N fertilizer treatments from 2016 to 2020 (%).

Treatment	2016	2017	2018	2019	2020	Average NUE
CRN60	66.3 ± 1.27	69.9 ± 1.14	69.8 ± 1.46	76.1 ± 0.81	72.0 ± 4.17	70.8 ± 0.58
CRN120	51.2 ± 1.23	54.3 ± 2.92	56.1 ± 3.18	56.1 ± 2.87	51.8 ± 3.23	53.9 ± 0.71
CRN180	42.3 ± 0.44	43.7 ± 1.61	48.3 ± 0.44	49.2 ± 0.20	41.9 ± 2.31	45.1 ± 0.69
CRN240	31.3 ± 0.89	33.0 ± 1.14	36.4 ± 0.95	36.4 ± 2.91	31.1 ± 1.94	33.6 ± 0.37
N60	66.0 ± 2.09	64.8 ± 1.93	65.6 ± 1.12	72.5 ± 1.43	62.3 ± 4.31	66.2 ± 0.36
N120	46.0 ± 0.41	50.2 ± 1.43	51.1 ± 1.03	48.6 ± 3.91	47.6 ± 2.90	48.7 ± 1.25
N180	36.3 ± 0.79	37.8 ± 0.53	41.5 ± 0.96	42.6 ± 0.72	38.1 ± 2.27	39.3 ± 0.53
N240	28.6 ± 0.40	29.6 ± 0.09	33.2 ± 0.88	34.5 ± 3.08	29.6 ± 2.36	31.1 ± 0.71
Type	**	**	**	**	*	**
Rate	**	**	**	**	**	**
Type × Rate	ns	ns	ns	ns	ns	ns

Statistically significant differences (**P<0.01; *P<0.05) and no significant differences *(P >*0.05, ns) are shown. Values are the means ± SEs (n = 3).

### 3.4 NH_3_ volatilization

**Figure 6:**
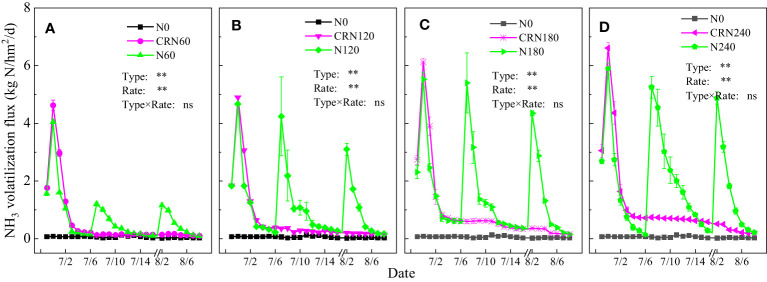
NH_3_ volatilization flux under the blended CRN treatments (red graph) and conventional N fertilizer treatments (green graph) with applied N rates of 0, 60, 120, 180, and 240 kg/hm^2^ in the 2019 rice season **(A–D)**.

The NH_3_ volatilization flux was affected by the rates and types of N fertilizers and their application methods. In 2019, the NH_3_ volatilization flux under conventional N fertilizer treatments and blended CRN treatments was 0.08–5.90 and 0.10–6.61 kg/hm^2^/d, with averages of 1.28 and 0.80 kg/hm^2^/d, respectively (**Figures 6A–D**). The NH_3_ volatilization flux peaked on the first to third day after N fertilizer application, followed by a rapid decline. The NH_3_ volatilization flux under conventional N fertilizer treatment was close to the background value 7 days after N fertilizer application. The NH_3_ volatilization flux in the blended CRN treatments was always higher than that in N0 during the rice growth period, and the increase was greater with the increase in N application. After basal fertilizer application at the same N rate, the NH_3_ volatilization flux increased under the blended CRN treatments compared with the conventional N fertilizer treatments, with average increases of 31.3%, 17.6%, 18.9%, and 32.9% at N rates of 60, 120, 180, and 240 kg/hm^2^, respectively. However, after tillering and panicle fertilization, the average decrease under the blended CRN treatments was 65.9% and 75.4%, 63.7% and 66.5%, 70.0% and 80.2%, and 79.7% and 77.6% at N rates of 60, 120, 180, and 240 kg/hm^2^, respectively, compared with that under the conventional N fertilizer treatments. Throughout the whole growing period of rice, the average reductions in the NH_3_ volatilization flux under the blended CRN treatments were 15.9%, 42.6%, 38.0%, and 40.7% compared with those under the conventional N fertilizer treatments (**Figures 6A–D**).

With an increase in the N rate, the cumulative NH_3_ emissions under different N fertilizer treatments gradually increased, but the ratio of NH_3_ emissions to the N application rate gradually decreased (**Table 3**). Cumulative NH_3_ emissions under conventional N fertilizer treatments ranged from 17.0 to 47.7 kg/km^2^, accounting for 19.9% to 28.3% of the N application rate. Cumulative NH_3_ emissions under CRN treatments ranged from 14.3 to 28.3 kg/km^2^, accounting for 11.8% to 23.8% of the N application rate. At the same N rate, the blended CRN treatments significantly increased the cumulative NH_3_ emissions compared with the conventional N fertilizer treatments after the basal fertilizer application. When the N rate was 60, 120, 180, and 240 kg/hm^2^, the cumulative NH_3_ emissions under the blended CRN treatment increased by 31.2%, 17.6%, 18.9%, and 32.9%, respectively. However, after tillering and panicle fertilization, the cumulative NH_3_ emissions under the blended CRN treatment decreased by 66.0% and 73.0%, 75.4% and 82.5%, 63.7% and 82.4%, and 66.5% and 81.7% at N rates of 60, 120, 180, and 240 kg/hm^2^, respectively. During the whole rice growth period, cumulative NH_3_ emissions under the blended CRN treatment decreased by 15.9%, 42.5%, 38.0%, and 40.7% at respective N rates of 60, 120, 180, and 240 kg/hm^2^.

**Table 3:** Cumulative NH_3_ emissions and the ratio of NH_3_ emissions under different N fertilizer treatments in the 2019 rice season.

Treatment	Basal fertilization		Tillering topdressing		Heading topdressing		Rice growing season	
	Cumulative NH_3_ emission kg/hm^2^	Ratio of NH_3_ emission %	Cumulative NH_3_emission kg/hm^2^	Ratio of NH_3_ emission %	Cumulative NH_3_ emission kg/hm^2^	Ratio of NH_3_ emission %	Cumulative NH_3_ emission kg/hm^2^	Ratio of NH_3_ emission %
N0	0.6 ± 0.05	–	0.7 ± 0.02	–	0.2 ± 0.01	–	1.5 ± 0.08	–
CRN60	11.8 ± 0.20	19.9 ± 0.33	1.5 ± 0.02	2.6 ± 0.03	0.9 ± 0.01	1.5 ± 0.01	14.3 ± 0.20	23.8 ± 0.33
CRN120	12.9 ± 0.15	10.7 ± 0.12	2.8 ± 0.21	2.4 ± 0.17	1.2 ± 0.00	1.0 ± 0.00	16.9 ± 0.34	14.1 ± 4.65
CRN180	17.0 ± 0.24	9.5 ± 0.13	5.4 ± 0.33	3.0 ± 0.18	1.7 ± 0.07	1.0 ± 0.04	24.1 ± 0.41	13.4 ± 2.99
CRN240	18.8 ± 0.64	7.9 ± 0.27	7.3 ± 0.05	3.1 ± 0.02	2.2 ± 0.03	1.0 ± 0.01	28.3 ± 0.07	11.8 ± 3.02
N60	9.0 ± 0.10	15.0 ± 0.17	4.5 ± 0.14	7.6 ± 0.24	3.45 ± 0.05	5.8 ± 0.09	17.0 ± 0.09	28.3 ± 0.16
N120	10.9 ± 0.01	9.1 ± 0.01	11.6 ± 2.76	9.6 ± 2.30	6.9 ± 0.13	5.8 ± 0.11	29.4 ± 2.63	24.5 ± 7.95
N180	14.3 ± 0.79	8.0 ± 0.44	14.9 ± 1.58	8.3 ± 0.88	9.8 ± 0.22	5.4 ± 0.12	38.9 ± 2.46	21.6 ± 5.01
N240	14.2 ± 0.21	5.9 ± 0.09	21.7 ± 0.83	9.0 ± 0.35	11.6 ± 0.08	4.9 ± 0.03	47.7 ± 0.92	19.9 ± 4.83
Type	**	**	**	**	**	**	**	**
Rate	**	**	**	ns	**	**	**	**
Type × Rate	*	**	**	ns	**	**	**	*

Statistically significant differences (**P<0.01; *P<0.05) and no significant differences *(P >*0.05, ns) are shown. Values are the means ± SEs (n = 3).

**Figure 7:**
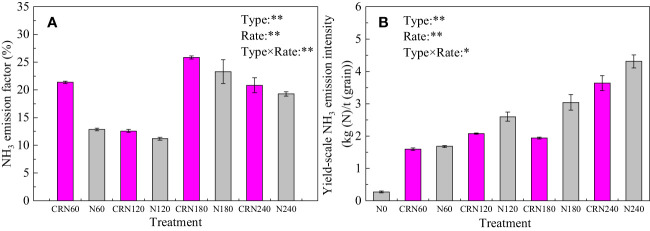
NH_3_ emission factors **(A)** and yield-scale NH_3_ emission intensity **(B)** under the blended CRN treatments (red graph) and conventional N fertilizer treatments (gray graph) with applied N rates of 0, 60, 120, 180, and 240 kg/hm^2^ in 2019.

The NH_3_ emission factors under different N fertilizer treatments gradually decreased with increasing N application rates (**Figure 7A**). The NH_3_ emission factors were 19.3%–25.9% and 11.2%–21.4% under conventional N fertilizer treatments and blended CRN treatments, respectively. At the same N rate, the NH_3_ emission factors under blended CRN treatments were significantly lower than those under conventional N fertilizer treatments (*P<*0.01). When the N rate was 60, 120, 180, and 240 kg/hm^2^, the NH_3_ emission factors decreased by 17.4%, 44.8%, 39.5%, and 42.0%, respectively.

Yield-scale NH_3_ emissions are driven by the effect of fertilizer management on both crop yields and NH_3_ emissions. With an increasing N application rate, yield-scale NH_3_ emissions gradually increased under the different N fertilizer treatments (**Figure 7B**). The yield-scale NH_3_ emissions were 1.6%–2.6% and 1.9%–3.0% under conventional N fertilizer treatments and blended CRN treatments, respectively. The blended CRN treatments significantly reduced these emissions compared with conventional N fertilizer treatments at the same N application rates of 60, 120, 180, and 240 kg/hm^2^ (*P<*0.01), with decreases of 17.9%, 44.8%, 42.7%, and 39.8%, respectively.

Pearson correlation analysis showed that rice yield was positively and significantly correlated with NH_3_ volatilization flux and cumulative NH_3_ emissions but negatively correlated with the NH_3_ emission factors (*P<*0.01; **Table 4**). NUE was negatively correlated with NH_3_ volatilization flux and cumulative NH_3_ emissions and significantly positively correlated with the NH_3_ emission factors and yield-scale NH_3_ emissions (*P<*0.05; **Table 4**).

**Table 4:** Pearson corrections among yield, NUE, cumulative NH_3_ emissions, NH_3_ emission factors, and yield-scale NH_3_ emission intensity in the 2019 rice season (n = 24).

Index	Yield	NUE	Cumulative NH3 emissions	NH3 emission factor	Yield-scale NH3 emission intensity
Yield	1	−0.726^**^	0.568^**^	−0.622^**^	0.395
NUE	−0.726^**^	1	−0.781^**^	0.450*	−0.721^**^
Cumulative NH3 emissions	0.568^**^	−0.781^**^	1	0.075	0.978^**^
NH3 emission factor	−0.622^**^	0.450*	0.075	1	0.218
Yield-scale NH3 emission intensity	0.395	−0.721^**^	0.978^**^	0.218	1

### 3.5 Economic benefit

**Figure 8:**
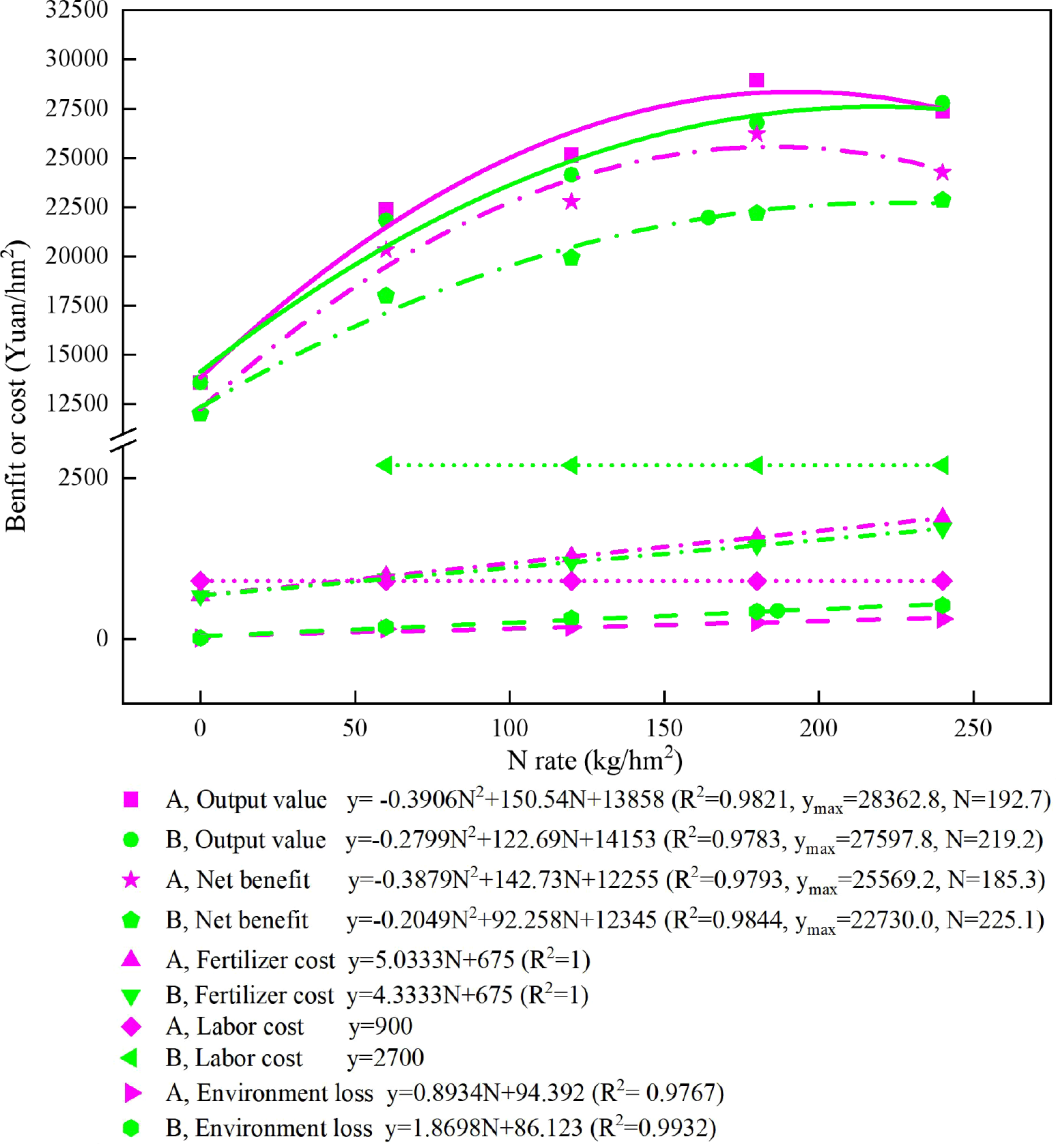
Output value, net benefit, fertilizer cost, labor cost, and environmental loss under the blended CRN treatments (red graph) and conventional N fertilizer treatments (green graph) with applied N rates of 0, 60, 120, 180, and 240 kg/hm^2^ in the 2019 rice season.

With the use of the economic benefit data for rice in 2019 as an example, fertilizer costs and environmental loss gradually increased with increasing N fertilizer application. CRN180 had the highest output value and net benefit of rice among all treatments (**Figure 8**). At the same N rate, the blended CRN treatments increased the fertilizer cost and net benefit compared with the conventional N fertilizer treatments; the specific increases were 42 and 2,329, 84 and 2,848, 126 and 4,026, and 168 and 1,412 yuan/hm^2^ at N applications of 60, 120, 180, and 240 kg/hm^2^, respectively. However, the environmental loss under the blended CRN treatments was lower than that under the conventional N fertilizer treatments at the same N application rates of 60, 120, 180, and 240 kg/hm^2^, with decreases of 30, 138, 171, and 214 yuan/hm^2^.

The relationship between output value, net benefit, and N application rate was simulated by the binary quadratic equation. When the net benefit of rice was the highest, the N application rate of the blended CRN treatment was 185 kg/hm^2^. In addition, the output value of rice was 25,569 yuan/hm^2^. The fertilizer cost and environmental loss were 1,608 and 260 yuan/hm^2^, respectively. The N application rate for conventional N treatment was 225 kg/hm^2^, with the highest net benefit. At the same time, the net benefit of rice was 22,730 yuan/hm^2^. The fertilizer cost and environmental loss were 1,650 and 507 yuan/hm^2^, respectively (**Figure 8**). Therefore, when the net benefit of rice was highest, the blended CRN treatment decreased the N fertilizer rate by 40 kg/hm^2^, increased the rice income by 2,839 yuan/hm^2^, and reduced the fertilizer cost and environmental loss by 43 and 247 yuan/hm^2^, compared with the conventional N treatment.

When the highest rice yield was achieved in 2019, the N rates of the blended CRN treatments and the conventional N fertilizer treatments were 193 and 219 kg/hm^2^, and the net benefit, fertilizer cost, and environmental loss were 25,548; 1,645; and 267 yuan/hm^2^ and 20,287; 1,634; and 496 yuan/hm^2^, respectively. When the corresponding rice yield under the two types of N fertilizer reached its maximum, the net benefit decreased, and the N application rate, fertilizer cost, and environmental loss increased compared with the highest net output of rice under these types of N fertilizer application.

## 4 Discussion

### 4.1 N release characteristics of CRNs

Temperature and moisture are important factors affecting the nutrient release of CRNs (Grant et al., 2012; Tong et al., 2018). Soil moisture was not a limiting factor for the N release of CRNs in paddy soil, which was generally in a flooded state. The average daily temperature during the rice season in 2019 was 28°C, which was 3°C higher than the temperature in the laboratory. Furthermore, the average daily temperature within 82 days after transplanting was higher than 25°C (**Figure 1**). As a result, the cumulative N release rate and the period during which N was released from CRNs with release periods of 40 and 90 days in paddy fields were higher than those in laboratory tests.

N is one of the main nutrients for rice growth. CRNs can provide N for rice growth by controlling the rate of N release (Zhang et al., 2018). Many researchers have used a single release period for CRNs throughout the whole rice growing period (Chen et al., 2020; Guo et al., 2019; Yu et al., 2013). In contrast, in this study, CRNs with different release periods were blended with conventional N for one-time fertilization. CRNs with release periods of 40 and 90 days played the roles of tillering fertilizer and panicle fertilizer, respectively, under conventional fertilization. Within 10 days after fertilization, the cumulative N release rate and the period during which N was released from the CRNs in these two release periods were low. The N required for rice growth was mainly supplied by conventional N during this period (**Figures 3A–D**). On the 14th day after fertilizer application, the cumulative N release rate of CRN1 with a release period of 40 days peaked to satisfy the tillering growth of rice (**Figures 3A, B**). On the 28th day after fertilizer application, the cumulative N release rate of CRN2, with a release period of 90 days, peaked. The cumulative N release rate of CRN2 decreased slowly from the 28th day to the 90th day, providing N for the booting, heading, and filling stages of rice (**Figures 3C, D**). The blended CRNs and normal N fertilizer treatments had similar biomass accumulation and N accumulation characteristics during the rice growth period in 2019 (**Figures S2A, B**, **S3A, B**). This indicated that blended CRNs with conventional N can achieve the effect of split fertilization with conventional N to meet the rice growth demand.

### 4.2 Blended CRN reduced NH_3_ volatilization

NH_3_ volatilization is one of the main methods of N loss in agricultural fields (He et al., 2018), and this loss can account for 10%–30% of the applied N rate (Yu et al., 2019). The NH_3_ volatilization loss in rice fields was closely related to the N rate and application time, which increased with an increased N application rate. This is mainly because an increase in the N rate greatly increases the NH_4_^+^-N concentration in field surface water (Yu et al., 2013). The results of this study are consistent with this finding (**Figures 6A–D**, **7A, B**; **Table 3**). However, coated controlled-release N fertilizer can retard the N dissolution process by blocking the movement of water into and out of the coated fertilizer, so that the NH_4_^+^-N concentration in the paddy soil and surface water is low (Li et al., 2018; Yang et al., 2019). Consequently, the ratio of NH_3_ emissions under the blended CRN treatments was lower than that under conventional N fertilizer treatments. Guo et al. (2018) found that there was no obvious peak of NH_3_ volatilization flux during the whole rice growth period after the one-time application of CRN as a basal fertilizer, which was consistently maintained at lower levels. Therefore, cumulative NH_3_ emissions under the one-time application of CRN were 57.7%–70.5% lower than those under conventional split N application in early and late rice at the same N rate (Guo et al., 2018). The effect of CRN on reducing NH_3_ volatilization loss was better than that in this study. NH_3_ volatilization loss was concentrated after basal fertilization under the blended CRN treatments in this study. Compared with the conventional N fertilizer treatments, the blended CRN treatments after basal fertilization increased the N application rate by 50% and the cumulative NH_3_ emissions by 17.6%–32.9%. However, after tillering and panicle fertilization, the cumulative NH_3_ emissions under the blended CRN treatments decreased by 63.7%–75.4% and 73.0%–82.5%, respectively. This result indicated that the contribution of blended CRNs to the reduction in NH_3_ volatilization loss was mainly after rice tillering.

### 4.3 The optimum economic application rate of blended CRNs

The relationship between the N application rate and rice yield is not a simple linear relationship. A linear plus platform model is commonly used to fit the relationship between crop yield and the N application rate (Alivelu et al., 2003; Li et al., 2015). A quadratic equation can also describe the relationship well (Kim et al., 2019; Ju et al., 2021). In this study, the results of five consecutive years of location experiments showed that the relationship between the N application rate and rice yield in both the blended CRN treatments and the conventional N fertilizer treatments could be simulated using a binary quadratic equation (**Figures 4A–E**, **5**). The maximum yield under the blended CRN treatments reached 8,295–11,347 kg/hm^2^, which was only 1.8%–5.6% higher than that under conventional N fertilizer treatments. When the controlled-release N fertilizer treatment reached its maximum yield, the corresponding N rate under the blended CRN treatments was 191–214 kg/hm^2^, which was 12.5% lower than the average N rate under conventional N fertilizer treatments. Li et al. (2015) showed that the N rate under the conventional N fertilizer treatments was 197 kg/hm^2^ when rice reached the maximum yield in Zhejiang Province using the linear plus platform method, i.e., the N rate was decreased by more than 32.9%. The results of this study showed that the N rate for the highest rice yield was like that reported in the study by Li et al. (2015), but the reduction in N was lower, mainly due to the higher N rate in Zhejiang Province, where the N rate can reach 360 kg/hm^2^.

Conventional split-application urea can result in high N losses through NH_3_ volatilization, runoff, and other ways, leading to a lower NUE compared with that of CRN applied one time (Chen et al., 2020; Guo et al., 2018; Li et al., 2015; Zhang et al., 2018). In this study, the results of five consecutive years of location experiments showed that the blended CRN treatments increased NUE by 6.9%–14.8% on average compared with the conventional N fertilizer treatments at the same N application rate. The N application rate had a significant effect on NUE, which was significantly higher at the low N rate than at the high N rate (**Table 2**; **Figure 5**).

Developing countries face the dual challenges of food security and environmental friendliness (Chen et al., 2011). Therefore, the Ministry of Agriculture of the People’s Republic of China (MOA) (2015) issued a policy toward a zero increase in fertilizer use. With the collaborative efforts of the government, scientific research departments, and rice growers under the guidance of the government, the N fertilizer rate in the Chaohu watershed is decreasing (Wu et al., 2020). However, the task of achieving high green high rice yields in the Chaohu watershed remains daunting. The economic benefit of rice production should also be considered (Emran et al., 2019).

In this study, among the nine treatments, the net benefit of the blended CRN treatments increased by 6.2%–18.1% compared with the N conventional fertilizer treatments, considering labor cost, fertilizer cost, and other environmental losses. CRN at a N rate of 180 kg/hm^2^ achieved the highest net benefit for rice. The NUE was 49.2%. A N rate of 180 kg/hm^2^ is also recommended for rice in the middle and lower Yangtze River basins (Liu et al., 2016). The N application rate under the blended CRN treatments was 193 kg/hm^2^, reaching the highest yield of rice. The corresponding NUE was 42.4%, which is 3.4 percentage points higher than that in the Chaohu watershed (Yu and Shi, 2015), which resulted in yield priority. Based on the binary quadratic equation to simulate the relationship between the N rate and net benefit of rice, it was found that the net benefit was the highest when the N rate under the blended CRN treatments was 185 kg/hm^2^. Under this condition, the NUE was 43.8%. The N rate and NUE were negatively correlated (**Figure 5**). Considering rice yield, environmental benefit, and economic benefit, the N application rate of blended CRNs was compared. The lowest N rate was achieved when the environmental benefit was prioritized, which could be used as the lower limit of the optimum N rate for blended CRNs. Prior to considering yield, the highest N rate could be used as the upper limit of the optimum N rate for blended CRNs. With priority consideration of the economic benefit, the N rate was between the lower and upper limits of the optimum N rate. The results for five consecutive years showed that the N rate under the blended CRN treatments was 191–214 kg/hm^2^, giving priority to yield consideration. Therefore, the optimum economic N rate of blended CRN was 180–214 kg/hm^2^ considering yield, environmental benefit, and economic benefit. Under the conventional N fertilizer treatments, the N rate was 212–278 kg/hm^2^, giving priority to yield consideration. Among the four conventional N fertilizer treatments, the N rate with the highest net benefit was 240 kg/hm^2^. Prior to considering the economic benefit, the N rate was 225 kg/hm^2^ under conventional N fertilizer treatments. Therefore, the optimum economic N rate of the conventional N fertilizers was 212–278 kg/hm^2^ considering yield, environmental benefit, and economic benefit. The optimum economic N rate of the blended CRN was significantly lower than that of conventional N fertilizer.

## 5 Conclusions

Urea-blended CRNs with a 4:3:3 ratio according to the N rate and release periods of 40 and 90 days adequately satisfied the N demand of rice during the whole growing period. Like the conventional N fertilizer treatments, the binary quadratic equation simulated the relationship between rice yield and N rate under the CRN treatments. The results showed that the N rate was 191–214 and 212–278 kg/hm^2^ for the highest yield under the blended CRN treatments and the conventional N fertilizer treatments, respectively, from 2016 to 2020. The rice net benefit of CNR180 was the highest among all treatments, and that of N240 was the highest among the four conventional N fertilizer treatments in 2019. NH_3_ volatilization increased with increasing N rates, but NUE decreased with increasing N rates. The blended CRN treatments reduced NH_3_ volatilization after tillering fertilizer and increased NUE compared with conventional N fertilizer treatments at the same N rate. Considering the yield output value, fertilizer cost, labor cost, and environmental loss, the optimum economic N rate was 180–214 kg/hm^2^ for the blended CRN treatments and 212–278 kg/hm^2^ for the conventional N fertilizer treatments in the Chaohu watershed in the Yangtze River Delta, China. Therefore, urea-blended CRNs are a promising measure to improve the NUE and economic income of rice because these CRNs can decrease NH_3_ volatilization and adverse environmental outcomes.

## Data availability statement

The raw data supporting the conclusions of this article will be made available by the authors, without undue reservation.

## Author contributions

MY: conceptualization, resources, data curation, and writing-original draft. YS: conceptualization and funding acquisition. GW: supervision and resources. JW: investigation and data curation. CL: methodology and software. TT: writing-review and editing. XZ: data curation. WW: visualization. YJ: sample measurement. All authors contributed to the article and approved the submitted version.

## Supplementary material

Supplementary Figure 1 The location and treatments of the experiment.

Supplementary Figure 2 Biomass accumulation under the blended CRN treatments **(A)** and conventional N fertilizer treatments **(B)** with applied N rates of 0, 60, 120, 180, and 240 kg/hm^2^ in the 2019 rice season.

Supplementary Figure 3 N accumulation under the blended CRN treatments **(A)** and conventional N fertilizer treatments **(B)** with applied N rates of 0, 60, 120, 180, and 240 kg/hm^2^ in the 2019 rice season.
