# Supplementary material for: The optimum economic nitrogen rate of blended controlled-release nitrogen fertilizer for rice in the Chanoyu watershed in the Yangtze River Delta, China
Source: Front Plant Sci. 2023 Apr 11;14:1144461. doi: 10.3389/fpls.2023.1144461 (PMC10126250; doi:10.3389/fpls.2023.1144461)
Supplement: Supplementary Figure 1 — The location and treatments of the experiment. [file Presentation_1.pdf]

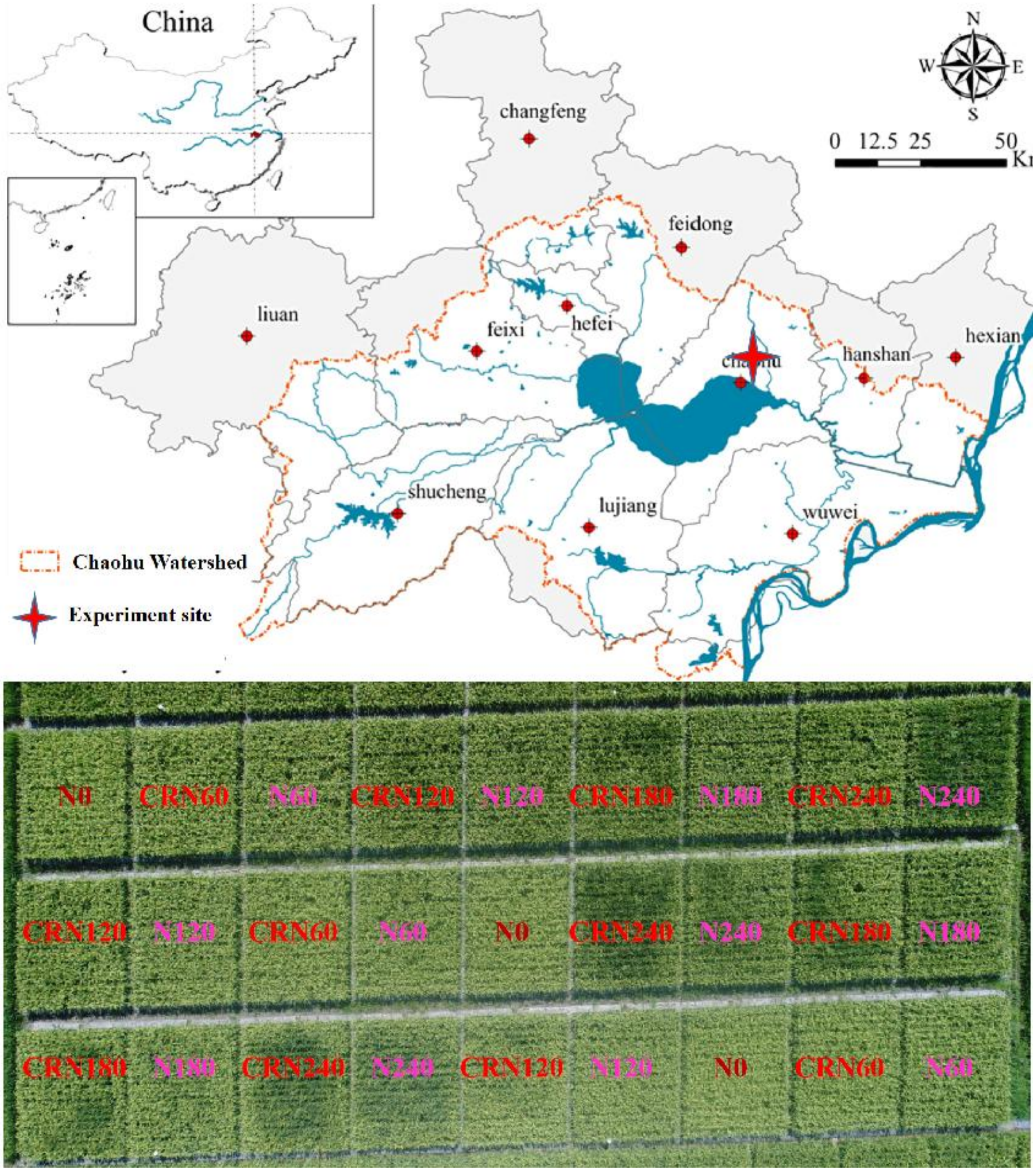

Supplementary Fig. 1 The location and treatments of the experiment.

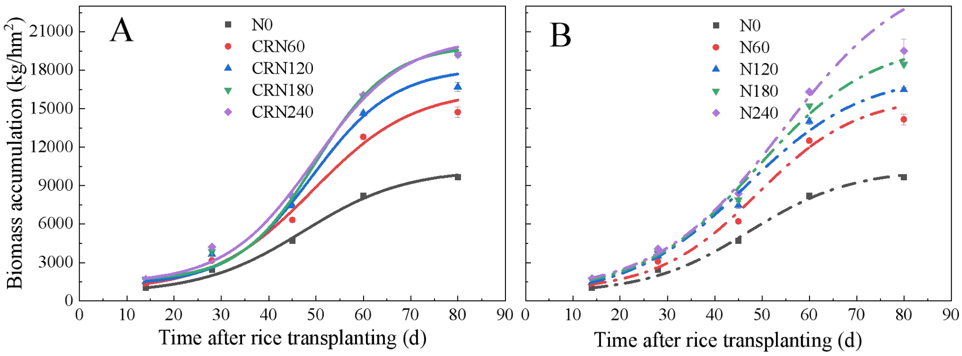

Supplementary Fig. 2 Biomass accumulation under the blended CRN treatments (A) and conventional N fertilizer treatments (B) with applied N rates of 0, 60, 120, 180, and 240 kg/hm^2 in the 2019 rice season.

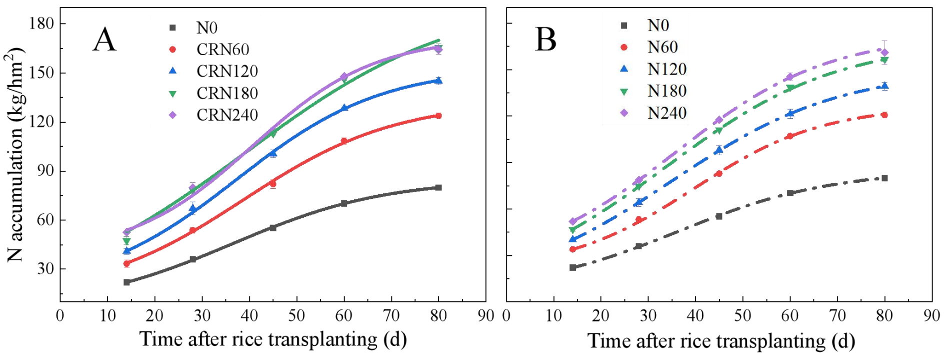

Supplementary Fig. 3 N accumulation under the blended CRN treatments (A) and conventional N fertilizer treatments (B) with applied N rates of 0, 60, 120, 180, and 240 kg/hm^2 in the 2019 rice season.
